# Introduction. Neuroeconomics: the promise and the profit

**DOI:** 10.1098/rstb.2008.0153

**Published:** 2008-10-01

**Authors:** Wolfram Schultz

**Affiliations:** Department of Physiology, Development and Neuroscience, University of CambridgeDowning Street, Cambridge CB2 3DY, UK

**Keywords:** reward, decision, risk, social, intention, neuron

## Abstract

Neuroeconomics investigates the neural mechanisms underlying decisions about rewarding or punishing outcomes (‘economic’ decisions). It combines the knowledge about the behavioural phenomena of economic decisions with the mechanistic explanatory power of neuroscience. Thus, it is about the neurobiological foundations of economic decision making. It is hoped that by ‘opening the box’ we can understand how decisions about gains and losses are directed by the brain of the individual decision maker. Perhaps we can even learn why some decisions are apparently paradoxical or pathological. The knowledge could be used to create situations that avoid suboptimal decisions and harm.

Recently, a colleague, Professor of Economics, remarked in a widely distributed email that neuroeconomics is about the silliest thing he could think of. Upon which another colleague, an understanding psychiatrist, replied that many neuroscientists assume that the brain controls the behaviour. This is the sort of argument and discussion one can also hear, with different levels of intensity, in the lecture halls and corridors of conferences. Who is right? Does neuroeconomics primarily produce amusing illustrations on what science can do with the world or does it help to solve certain issues in economics and neuroscience? And what do the neuroscientists think after they have recovered from the shock that yet another discipline enters the picture while they are trying to understand the functions and mechanisms of the human and animal brain (and mind)?

When neuroeconomics started less than 10 years ago, the nicely coherent economic utility theory with its more than 200 years of tradition had shown serious cracks in its power of explanation incurred by inconsistent behavioural preferences and so-called irrational decisions. Prospect theory provided explanations for some of these problems but still lacked a coherent framework. The contentious issues might be resolved, and a new economic decision theory emerge, by investigating the brains of decision makers. The hope was that investigations of biological mechanisms could help to discard those alternatives for which there was no neural mechanism. The promise might be exaggerated but it helped to drive the field into a flurry of human brain imaging and animal neurophysiological studies investigating the neural basis of economic decision making. What neuroeconomics is less likely to provide is an explanation for economic phenomena beyond the influence of the individual decision maker, including market forces and the laws of supply and demand. However, even these safe fields outside the reach of heretic neuroeconomics might be affected, as demands can be manipulated and might depend, among other factors, on the brains of individuals making decisions.

In contrast to the economists, the neuroscientists had few problems with neuroeconomics and were quick to use its potential for future studies. Neuroeconomics builds on behavioural economics, and behavioural imaging and neurophysiological studies have an insatiable appetite for controlled and quantifiable behavioural tasks. Any addition to the repertoire of behavioural paradigms is welcome, even more so when they come packaged into neat theories that appear, at first sight, consistent with measurable evidence. Despite the wonderful tests and theories developed over more than a hundred years by experimental psychologists, the tasks emerging from behavioural economics allowed neurophysiologists to connect behavioural phenomena that seemed far apart and provided long-desired explanations and quantitation through surprisingly simple and intuitive tests. In addition, behavioural game theory allowed neuroscience to move into the domain of controlled and quantifiable social behaviour. No wonder neuroscientists had no problems adopting neuroeconomic thoughts and paradigms.

This special issue presents examples of current work in neuroeconomics in order to convey some of the enthusiasm and insights for economic and neuroscience research. Informed decision making requires the neural coding of basic decision parameters about predictive information on future outcomes and their uncertainty (risk and ambiguity), both for gains and losses. The first three contributions treat exactly these issues in humans and animals. Knutson & Greer (2008) review studies that provide insights into brain mechanisms engaged with the anticipation of gains and losses, in particular the striatum and insula. However, primary aversive biological outcomes such as electrical shocks need to be investigated separately from the more conditioned nature of monetary losses, and the fear induced by shocks impacts powerfully on decisions and illustrates the role of negative emotions in decision making. The issue is taken up in the paper by Delgado *et al*. (2008), who investigate the basic mechanisms underlying predictive aversive learning, notably aversive prediction errors. The contribution by Schultz *et al*. (2008) describes the discovery of risk and ambiguity signals in the human and animal brain. These signals do not only inform the individual about the incompleteness of information about outcomes but could also modulate the perceived value of outcomes according to financial decision theory. For example, risk avoiders attribute less value to outcomes that are less certain. These data illustrate how assumptions of specific economic theories, such as the mean variance approach of finances, may be related to neurobiological mechanisms, thus putting a neurobiological basis to certain economic theories. The studies also demonstrate how such neurobiological data become interpretable through economic theories, thus helping to understand brain function.

Once we advanced our understanding of the neural coding of basic decision parameters, including uncertainty, we need to consider other key factors contributing to decisions, notably temporal delays, and then put the data into the larger perspective. Outcomes that occur later have less subjective value for the decision maker, maybe because they appear less certain. The contribution by Rick & Loewenstein (2008) argues for a specific appearance of uncertainty in distant outcomes. Later outcomes are less tangible for the decision maker who therefore assigns less value to them. However, these decision parameters are not set in stone. Rather, outcomes are valued according to the very simple rule of survival and competition in the face of scarce nutritional sources, and the capacity to do this in a satisfactory manner ultimately determines the survival of the species (or rather its genes) during biological evolution. This is the perspective that may help us to understand why preferences and risk attitudes shift, and our neural concepts need to accommodate such changes. The contribution by Watson & Platt (2008) addresses the issue and provides examples of brain mechanisms that can be best understood on the basis of theories of behavioural ecology. Taken together, these reviews describe the properties and constraints of basic economic decision parameters and attempt to outline the functions of brain systems that have evolved to deal with these crucial biological functions.

One of the most novel contributions of behavioural economics and game theory to neuroscience is to provide a quantifiable handle on social processes. We are privileged to have several such contributions dealing with behaviour, human imaging and neurophysiology. The contribution by Lakshminaryanan *et al*. (2008) describes how monkeys make the same biased decisions that are typical for humans and which gave rise to prospect theory, namely the endowment effect. In addition, monkeys can use tokens for exchanging edible goods. These data indicate that paradoxical choice phenomena not covered by utility theory and the use of abstract currency exist also in non-human animals, suggesting similar neural correlates. The contribution by Seo & Lee (2008) uses formal games in monkeys against a computer to study the role of reinforcement on game performance and to assess the contribution of specific cortical areas to the critical behavioural components of game performance. Prefrontal neurons track previous movement choices and reward history, whereas anterior cingulate tracks primarily reward history. These data are compatible with previously known physiological functions of these regions and demonstrate how novel behavioural situations such as formal games allow us to understand how specific cortical areas contribute to richer but nevertheless well-controlled behavioural situations than previously tested in laboratory settings. The contributions by Frith & Singer (2008) and by Krueger *et al*. (2008) carry the investigations of formal games from monkeys to humans. They come with the different but convergent perspectives of behavioural game theory and cognitive psychology, respectively. They review various games such as Prisoner's dilemma, dictator and ultimatum games and trust and investment games and describe the social components of performance in these games such as cooperation, defection, social distance, sympathy and empathy, trust, fairness, resentment of unfair offers, reciprocation, anger and disgust and altruistic punishment. The brain processes underlying social interactions tested by game playing involve reinforcement with prediction error coding, representation of sensations and emotions in others, and mentalizing of the other player's intentions. They identify the corresponding neural correlates in the striatum (reward prediction error), insula (empathy and resentment of unfair treatment), orbitofrontal cortex (cooperation) and medial frontal cortex (mentalizing). Taken together, the game studies provide us with excellent examples of brain mechanisms underlying decisions in the social domain.

We hope you will have fun reading these papers, appreciate the promise of neuroeconomics and profit from the insights emerging from this amazing new avenue of research. These are exciting times for both economists and neuroscientists.
